# Spectrum and characteristics of germline *PALB2* pathogenic variants in 1556 early-onset breast cancer patients in China

**DOI:** 10.1007/s00432-024-05758-7

**Published:** 2024-06-25

**Authors:** Jing Li, Peng He, Qindong Cai, Lili Chen, Yali Wang, Weifeng Cai, Yibin Qiu, Shunyi Liu, Wenhui Guo, Minyan Chen, Yuxiang Lin, Chuan Wang, Fangmeng Fu

**Affiliations:** 1 Department of Breast Surgery, Fujian Medical University Union Hospital, Fuzhou, 350001 Fujian Province China; 2 Department of General Surgery, Fujian Medical University Union Hospital, Fuzhou, 350001 Fujian Province China; 3 Breast Cancer Institute, Fujian Medical University, Fuzhou, 350001 Fujian Province China

**Keywords:** *PALB2* variants, Germline mutation, Genetic sequencing, Early-onset breast cancer, China

## Abstract

**Purpose:**

Limited data are available regarding the partner and localizer of *BRCA2* (*PALB2*) in Chinese patients with early breast cancer. This study aimed to assess the spectrum and characteristics of germline *PALB2* pathogenic variants in this population.

**Methods:**

Peripheral blood samples were collected from 1556 patients diagnosed with *BRCA1/2*-negative early-onset breast cancer*.* All coding regions and exon‒intron boundaries of the *PALB2* genes were screened through next-generation sequencing.

**Results:**

The prevalence of *PALB2* pathogenic variants was approximately 0.77% in the cohort. Eleven *PALB2* pathogenic variants were identified in twelve participants, including five frameshift mutations and six nonsense mutations. All other variants were detected once, except for *PALB2* c.1056_1057del (detected twice). Two *PALB2* carriers (2/12, 16.7%) have documented family history of breast cancer and/or ovarian cancer. Patients with a positive family history exhibited a threefold higher possibility of being identified as *PALB2* carriers than those without a family history (2% vs. 0.69%), although the difference was not statistically significant (*p* = 0.178). Compared to non-carriers, *PALB2* carriers has a tendency to appear in younger age (≤ 30 years) (25% vs 14.4%), human epidermal growth factor receptor-2 (HER2)-negative status (83.3% vs. 70.2%), and diagnosed with invasive micropapillary carcinoma (16.7% vs 3.1%).

**Conclusion:**

The prevalence of the germline *PALB2* pathogenic variants was approximately 0.77% in Chinese patients with *BRCA1/2*-negative early-onset breast cancer. Our findings is crucial for understanding population-specific genetic risks and offering insights that can enhance genetic counseling and genetic testing strategies in this population.

## Introduction

The partner and localizer of *BRCA2* (*PALB2*) is a crucial mediator of homologous recombination-mediated DNA repair. During DNA double-strand breaks (DSBs), *BRCA1* recruits *PALB2*, which subsequently binds to *BRCA2*. This binding localizes *BRCA2* and radiation-sensitive protein 51 (*RAD51*) to damaged DNA sites, facilitating recombination repair, thereby contributing to the preservation of genome integrity and the suppression of cancer development (Xia et al. 2006; Zhang et al. 2009; Sy et al. 2009; Buisson et al. 2010). Germline *PALB2* variants are associated with a moderate-to-high risk of development breast cancer (Easton et al. 2015; Couch et al. 2017; Zhou et al. 2020; Breast Cancer Association Consortium et al. 2021), specifically in younger carriers (Antoniou et al. 2014). Individuals aged over 40 years carrying the *PALB2* gene exhibit a 5–8 times higher risk of breast cancer compared to the general population, whereas *PALB2* carriers aged below 40 years exhibit an 8–9 times higher risk than the general population (Antoniou et al. 2014).

The prevalence of germline *PALB2* pathogenic variants has been identified in 0.66–0.97% of Chinese patients with breast cancer unselected for predisposing factors (Zhang et al. 2017; Wu et al. 2020; Zhou et al. 2020; Fu et al. 2021), 0.40%–0.86% in other Asian cohorts (Japan (Momozawa et al. 2018) and Malaysia (Yang et al. 2017)), and 0.87% in Caucasian population (Couch et al. 2017). Among patients with hereditary high-risk breast cancer, including early-onset breast cancer, familial breast cancer, bilateral breast cancer, triple-negative breast cancer (TNBC) and male breast cancer, *PALB2* mutation rates are relatively high. In the European population, rates range from 1.3 to 2.7% (Butz et al. 2023; Couch et al. 2015; Erkko et al. 2007; Kluska et al. 2017; Weitzel et al. 2019), 1.0–1.3% in the Australian and New Zealand populations (Southey et al. 2010; Thompson et al. 2015), and 1.0%–2.6% in the previous Chinese studies (Cao et al. 2009; Li et al. 2015; Kwong et al. 2021). The results of mutation frequency of *PALB2* vary due to different criteria for hereditary high-risk factors.

Breast cancer is the most common cancer among Chinese women, with a reported crude incidence of 52.81 per 100,000 in 2019 (Yin et al. 2023). This includes more than 16% of early-onset breast cancers, with a rising trend (Guo et al. 2019; Yin et al. 2023). A substantial number of early-onset cases pose a significant public health burden in China. Early onset of breast cancer is a hereditary high-risk factor and associated with unique clinicopathological characteristics. Identification of *PALB2* variants not only enhances disease management for carriers but also offers valuable data for genetic risk assessment in other family members. However, numerous studies on *PALB2* germline pathogenic variants in early-onset breast cancer have been focused on Europeans and Americans, with limited data available on young Chinese patients.

Analysis of the genetics in a large-scale early-onset breast cancer cohort will offer a deeper insight into the distribution and characteristics of *PALB2* variants within this group. This study aimed to assess the spectrum and clinicopathological features of *PALB2* pathogenic variants in Chinese patients with early-onset breast cancer, irrespective of family history.

## Material and methods

### Study population

The cohort included individuals with a confirmed invasive breast cancer diagnosis at or below the age of 40 years at the Fujian Medical University Union Hospital, a tertiary care hospital in China, between 2005 and 2023. All the individuals tested negative for germline *BRCA1/2* mutations. Male patients, individuals under age 18 years old, or those with carcinoma in situ were excluded. The final analysis included 1556 eligible patients with early-onset breast cancer. Peripheral blood specimens (3–5 ml) were collected after obtaining informed consent. In this study, the procedures involving human participants adhered to the ethical standards of the institutional and/or national research committee and the Declaration of Helsinki and its subsequent amendments or comparable ethical standards.

### Data collection

Demographic data were collected using semi-structured questionnaires, and clinicopathological data were extracted from electronic medical records by uniformly trained medical staff. Demographic data included prior diagnosis of invasive breast cancer, personal history of breast cancer and family history of cancer. Clinicopathological characteristics included tumor size, lymph node involvement, morphology, histological grade, estrogen receptor (ER), progesterone receptor (PR), human epidermal growth factor receptor 2 (HER2), and Ki67 index. ER- and PR-positive status was defined as > 10% tumor cell staining. Hormone receptor (HR) -positive status was defined as having positive status for ER or/and PR. HER2 positive status was defined as HER2 protein expression of 3 + or HER2 gene amplification by fluorescence in situ hybridization (FISH).

### DNA extraction and sequencing of gene *PALB2*

Genomic DNA was extracted from peripheral blood samples using the Whole Blood Genome DNA Isolation Kit (Bioteke, Beijing, China) following the instructions provided by the manufacturer. The purity and concentration of DNA were measured on a NanoDrop2000 spectrophotometer (Thermo Fisher Scientific, USA) and quality of DNA was checked by agarose gel electrophoresis.

Germline DNA were sequenced for the *PALB2* gene as part of a multigene custom panel. *PALB2* gene fragments were amplified by multiplex PCR. All coding regions and exon–intron boundaries of *PALB2* were sequenced by next-generation sequencing (NGS) on the Illumina Novaseq platform (Illumina, CA, USA), conducted by the AITA Biomedical Research Institute (Shanghai, China) and AmoyDx Biomedical Technology Co., Ltd (Xiamen, China). A minimum coverage depth of 300 × , a mean coverage depth of 1000 × , uniformity of 90%, and variant allele frequencies (VAFs) of 20% were set as cutoff values in this study. Additionally, the proportion of Q30 bases was required to be higher than 75%. The sequencing results were aligned to the human reference sequence *PALB2* (NM_024675.3) using the Burrows-Wheeler Aligner (BWA) tool. Base quality score recalibration, indel realignment, and variant calling were performed using the Genome Analysis Toolkit (GATK). All the variants were annotated using ANNOVAR (http://www.openbioinformatics.org/annovar/) and validated though Sanger sequencing.

Variants were named according to the of Human Genome Variation Society (HGVS) Nomenclature recommendations (https://hgvs-nomenclature.org/) and classified based on the American College of Medical Genetics and Genomics/Association for Molecular Pathology (ACMG/AMP) guidelines (Richards et al. 2015) and ClinVar (https://www.ncbi.nlm.nih.gov/clinvar/). Additionally, this study included both pathogenic and likely pathogenic variants.

### Statistical analysis

The study compared continuous variables between *PALB2* pathogenic variant carriers and non-carriers using the Mann–Whitney U test, expressing the differences as median and interquartile range (IQR). The differences in categorical variables were analyzed as frequencies (%) using Fisher’s exact test. *P* values of < 0.05 were considered to indicate statistical significance. All of the statistical analyses were conducted using the R software version 4.3.0.

## Results

### Characteristics of patients with early-onset breast cancer

The characteristics of the 1556 participants in this cohort are display in Table 1. The median age of the participants was 36 years (range, 33–38 years). Of these, 225 patients (14.5%) were diagnosed with breast cancer at the age of ≤ 30 years, 100 patients (6.4%) had a family history of breast cancer and/or ovarian cancer in a first- or second-degree relatives, and 50 patients (3.2%) were diagnosed with bilateral breast cancer at enrollment. HER2-positive status was observed in 29.7% of the patients. The majority of the molecular subtypes were HR+/HER2- breast cancer (54.3%), whereas the other three types of breast cancer had similar proportions, ranging from 14.5 to 16.0%. Invasive ductal carcinoma was diagnosed in 1235 patients (83.3%).

**Table 1:** Characteristic of overall population and comparisons between non-carriers vs. *PALB2* carriers

Variable n (%)	All cases (n = 1556)	Non-*PALB2* (n = 1544)	*PALB2* carriers		*p*
			(n = 12)	Prevalence %	
Age at first BC diagnosis, years					
Median age (range)	36 (33.0–38.0)	36 (33.0–38.0)	35 (31.50–37.25)	–	0.496
≤ 30	225 (14.5)	222 (14.4)	3 (25.0)	1.33	0.398
> 30	1331 (85.5)	1322 (85.6)	9 (75.0)	0.68	
FH of BC/OC					0.178
None	1456 (93.6)	1446 (93.7)	10 (83.3)	0.69	
Yes	100 (6.4)	98 (6.3)	2 (16.7)	2.00	
Bilateral BC					1.000
No	1506 (96.8)	1494 (96.8)	12 (100.0)	0.80	
Yes	50 (3.2)	50 (3.2)	0 (0)	0	
ER status					0.762
Positive	1062 (68.3)	1053 (68.2)	9 (75.0)	0.85	
Negative	494 (31.7)	491 (31.8)	3 (25.0)	0.61	
PR status					0.771
Positive	909 (58.4)	901 (58.4)	8 (66.7)	0.88	
Negative	647 (41.6)	643 (41.6)	4 (33.3)	0.62	
HR status					1.000
Positive	1085 (69.7)	1076 (69.7)	9 (75.0)	0.83	
Negative	471 (30.3)	468 (30.3)	3 (25.0)	0.64	
HER2 status					0.527
Positive	455 (29.7)	453 (29.8)	2 (16.7)	0.44	
Negative	1078 (70.3)	1068 (70.2)	10 (83.3)	0.93	
Unknown	23	23	0		
TNBC					0.703
Yes	245 (16.0)	244 (16.0)	1 (8.3)	0.41	
No	1288 (84.0)	1277 (84.0)	11 (91.7)	0.85	
Unknown	23	23	0		
Molecular subtypes					0.455
HR + /HER2-	833 (54.3)	824 (54.2)	9 (75.0)	1.08	
HR + /HER2 +	232 (15.1)	232 (15.3)	0 (0)	0	
HR–/HER2 +	223 (14.5)	221 (14.5)	2 (16.7)	0.90	
HR–/HER2-	245 (16.0)	244 (16.0)	1 (8.3)	0.41	
Unknown	23	23	0		
Ki-67					0.741
< 20%	370 (25.0)	368 (25.1)	2 (16.7)	0.54	
≥ 20%	1108 (75.0)	1098 (74.9)	10 (83.3)	0.90	
Unknown	78	78	0		
Tumor size					0.4
≤ 2 cm	720 (46.5)	716 (46.6)	4 (33.3)	0.56	
> 2 cm	827 (53.5)	819 (53.4)	8 (66.7)	0.97	
Unknown	9	9	0		
Lymph node status					0.146
Negative	761 (48.9)	758 (49.1)	3 (25.0)	0.39	
Positive	795 (51.11)	786 (50.9)	9 (75.0)	1.13	
Nuclear Grade					1.000
I + II	959 (65.8)	952 (65.8)	7 (63.6)	0.73	
III	498 (34.2)	494 (34.2)	4 (36.4)	0.80	
UNKNOWN	98	97	1		
Morphology					0.080
IDS	1235 (83.3)	1226 (83.3)	9 (75.0)	0.73	
IMPC	47 (3.2)	45 (3.1)	2 (16.7)	4.26	
Others	201(13.6)	200 (13.6)	1 (8.3)	2.17	
Unknown	73	73	0		

*BC* breast cancer; *FH* family history; *OC* ovarian cancer; *HER2* human epidermal growth factor receptor-2; *ER* estrogen receptor; *PR* progesterone receptor; *HR* hormone receptor; *TNBC* triple-negative breast cancer; *IDC* invasive ductal carcinoma; *PMC* pure mucinous carcinoma; *IMPC* invasive micropapillary carcinoma (pure or mix with invasive ductal carcinoma)

### *PALB2* pathogenic variant identified in this cohort

**Fig. 1:**
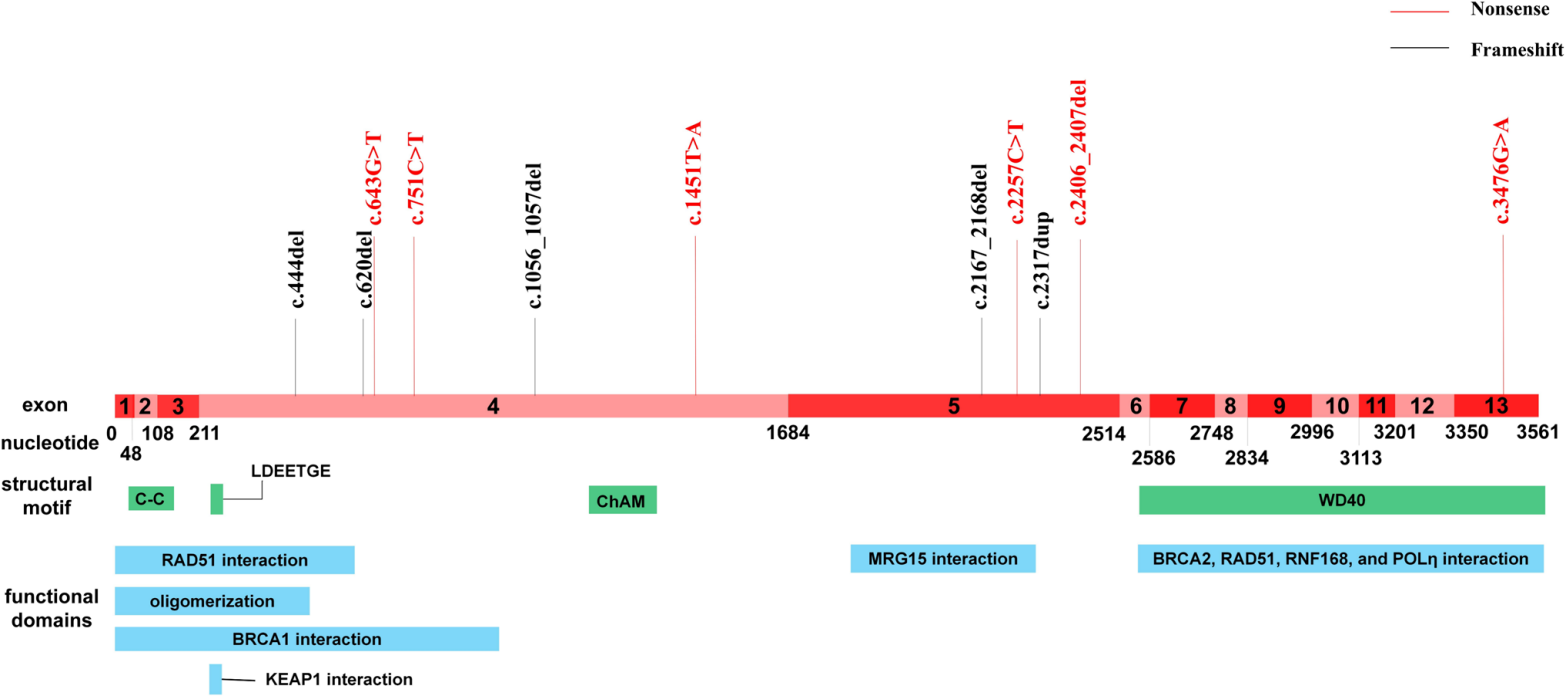
Schematic diagram of pathogenic *PALB2* variants detected in this cohort of early-onset breast cancer. *PALB2* comprises 13 exons and all the pathogenic variants are marked up in the schematic diagram of the *PALB2* coding sequence. Structural motifs and functional domains are also indicated. Red: nonsense; black: frameshift

Eleven *PALB2* pathogenic variants were identified in twelve patients, with a mutation rate of 0.77%. All variants were protein-truncation variants and had been previously reported (Table 2, Figure 1). Hotspot mutation regions were located in exon 4 (58.3%, 7/12) and exon 5(33.3%, 4/12). Among these, 50% were frameshift mutations, including c.1056_1057del (twice identified), c.444del, c.620del, c.2317dupA, and c.2167_2168del. The remaining 50% were nonsense mutations, including c.643G > T, c.751C > T, c.1451T > A, c.2257C > T, c.2406_2407del, and c.3476G > A. The *PALB2* c.3476G>A mutation resulted in an amino acid change within the WD40 domain, a functional domain of *PALB2* in the C-terminus. The *PALB2* c.444del, c.620del, c.643G>T and c.751C>T occurred within the *PALB2* oligomerization, *RAD51-* and *BRCA1-*binding domain, which located in the N-terminus of *PALB2* (Figure 1).

**Table 2:** Germline *PALB2* pathogenic variants detected in this early-onset breast cancer cohort

Exon	Variants	Amino Acid change	Mutation type	Classification	Case
4	c.444del	p.Lys149SerfsTer28	Frameshift	P/LP	1
4	c.620del	p.Pro207GlnfsTer16	Frameshift	P/LP	1
4	c.643G > T	p.Glu215Ter	Nonsense	P	1
4	c.751C > T	p.Gln251Ter	Nonsense	P	1
4	c.1056_1057del	p.Lys353IlefsTer7	Frameshift	P	2
4	c.1451 T > A	p.Leu484Ter	Nonsense	P/LP	1
5	c.2167_2168del	p.Met723ValfsTer21	Frameshift	P	1
5	c.2257C > T	p.Arg753Ter	Nonsense	P	1
5	c.2317dup	p.Thr773AsnfsTer2	Frameshift	P	1
5	c.2406_2407del	p.Cys802_Asp803delinsTer	Nonsense	P	1
13	c.3476G > A	p.Trp1159Ter	Nonsense	P	1

*P* pathogenic; *LP* likely pathogenic

### Characteristics of patients with *PALB2* pathogenic variants

Among the patients with germline *PALB2* pathogenic variants, the median age was 35 years compared to 36 years in the non-carriers (*p* = 0.496). *PALB2* carriers were more frequently observed in cases ≤ 30 years of age compared with non-carriers (25% vs 14.4%, *p* = 0.398). The prevalence of *PALB2* carriers among patients aged ≤ 30 years was approximately double that in patients aged 31–40 years (1.33% vs. 0.68%) (Table 1). Two patients with *PALB2* pathogenic variants had positive family histories: one with a history of breast cancer in her mother, and the other with a history of breast cancer in her aunt (Table 3). Patients with *PALB2* pathogenic variants were more likely to have a positive family history of breast cancer and/or ovarian cancer than non-carriers (16.7% vs. 6.3%). However, this difference was not statistically significant (*p* = 0.178). The mutation rate of *PALB2* in patients with a positive family history was 2%, which was 3-fold higher than that in patients without a family history (0.69%). None of the *PALB2* carriers were diagnosed with bilateral breast cancer.

**Table 3:** Characteristic of the 12 patients with *PALB2* pathogenic variants

Variants	Dx	FH with BC/OC	HR status	HER2 status	Grade	Histology	Metastatic LN
c.444del	35	None	–	–	III	IDC	Yes
c.620del	38	None	+	–	II	MUC	No
c.643G > T	32	None	+	–	II	IDC	Yes
c.751C > T	35	None	+	–	III	IDC	Yes
c.1056_1057del	36	None	–	+	unk	IDC	No
c.1056_1057del	40	Mother (BC)	+	–	II	IDC	Yes
c.1451 T > A	37	None	+	–	II	IMPC	Yes
c.2167_2168del	29	None	+	–	III	IMPC	Yes
c.2257C > T	29	None	–	+	III	IDC	Yes
c.2317dup	30	An aunt (BC)	+	–	II	IDC	Yes
c.2406_2407del	32	None	+	–	II	IDC	No
c.3476G > A	40	None	+	–	II	IDC	Yes

*Dx* age at initial diagnosis of breast caner; *FH* family history; *BC* breast cancer; *OC* ovarian cancer; *HR* hormone receptor; *HER2* human epidermal growth factor receptor-2; *TNBC* triple-negative breast cancer; *unk* unknown; *IDC* invasive ductal carcinoma; *IMPC* invasive micropapillary carcinoma mix with invasive ductal carcinoma; *MUC* mucinous carcinoma; *LN* lymph node

The *PALB2* carriers were predominantly HR-positive (75%, 9/12), and HER2-negative (83.3%, 10/12) breast cancer. Patients with HER2-/HR+ breast cancer exhibited the highest *PALB2* pathogenic variant prevalence at 1.08% (9/833), constituting 75.0% (9/12) of all *PALB2* carriers. In contrast, HER2-/HR- breast cancer, also known as triple-negative breast cancer (TNBC), exhibited a prevalence of 0.41%, representing 8.3% (1/12) of *PALB2* carriers. However, the differences in the distribution of the tumor Immunohistochemical characteristics between *PALB2* carriers and non-carriers were not statistically significant (Table 1 and Table 3).

Compared with non-carriers, *PALB2* carriers exhibited a slightly higher Ki-67 index (83.3% vs. 74.9%, *p* = 0.741), larger tumor size (66.7% vs. 53.4%, *p* = 0.4), and lymph node metastasis (75.0% vs. 50.9%, *p* = 0.146). Invasive ductal carcinoma was observed in 75% (9/12) of *PALB2* carriers compared with 83.3% of the non-carriers. Two carriers (16.7%) were diagnosed with invasive micropapillary carcinoma (IMPC) mix with invasive ductal carcinoma, and one carrier (8.3%) was diagnosed with mucinous carcinoma (Table 1 and Table 3). IMPC exhibited the highest mutation rate (4.26%) in *PALB2*, whereas the mutation rate in invasive ductal carcinoma was 0.73%. The difference in tumor morphology distribution was close to statistical significance (*p* = 0.080, Table 1).

## Discussion

Given the diverse spectrum of *PALB2* variants across ethnicities and regions, it is crucial to investigate the spectrum of *PALB2* variants in Chinese patients with early-onset breast cancer. In this study, we identified 12 carriers of *PALB2* pathogenic variants in 1556 Chinese patients with *BRCA1/2*-negative early-onset breast cancer, resulting in a mutation frequency of 0.77%. This represents the largest study to date on the spectrum and characteristics of germline *PALB2* pathogenic variants in this population.

Certain recurrent mutations identified as founder mutations significantly affect the prevalence in certain populations. *PALB2* c.2257C>T serves as a founder mutation in Greek populations (Vagena et al. 2019), while c.2167_2168del is a founder mutation in Italian and Hispanic populations (Catucci et al. 2016). These two mutations have been reported as recurrent mutations in unselected breast cancer studies in China (c.2257C>T: 6/16501 and 4/7657, respectively; c.2167_2168del: 12/16501 and 2/7657, respectively) (Deng et al. 2019; Zhou et al. 2020), however, we identified these mutations only once in each case. Although the *PALB2* c.509_510del and c.172_175del were identified as founder or hotspot mutations in European populations (Rogoża-Janiszewska et al. 2020), and *PALB2* c.2968G>T was found to be a hotspot mutation in women with breast cancer from Malaysia and Singapore, none of these were observed in our study (Ng et al. 2022). This highlights the ethnic and regional disparities in the spectrum of *PALB2* pathogenic variants.

*PALB2* c.1056_1057del was identified in two individuals, while the other variants were identified in a single individual. The mutation frequency of c.1056_1057del was 0.13% (2/1556), which is significantly higher than previous studies in unselected breast cancer in China (1/16501, 1/7657 and 0/2769, respectively) (Deng et al. 2019; Wu et al. 2020; Zhou et al. 2020) and other Asian countries (1/7840 in Malaysia and Singapore, and 0/7051 in Japan) (Momozawa et al. 2018; Ng et al. 2022). While *PALB2* c.751C > T was reported as a hotspot mutation in previous Chinese studies (Deng et al. 2019; Wu et al. 2020; Zhou et al. 2020). The frequency of c.751C > T was 0.1–0.33% in the unselected breast cancer cohort and 0.55% in a hereditary high-risk breast cancer cohort (Cao et al. 2009), which was higher than that in our early-onset breast cancer cohort (< 0.1%, 1/1556). Moreover, other hotspot mutations in Chinese unselected breast cancer, including c.1317del and c.3114-1G>A (Wu et al. 2020; Zhou et al. 2020), were not identified in our study. This observation may suggest a difference in the spectrum of *PALB2* pathogenic variants between early-onset breast cancer and other breast cancers in China. However, it could also be attributed to the low mutation rate of *PALB2* and the limited number of carriers detected in our study.

In our study, the prevalence of pathogenic variants in *PALB2* was 0.77%, which was higher than 0.66–0.71% observed in most large-scale cohorts of Chinese unselected breast cancer patients (Zhang et al. 2017; Wu et al. 2020; Fu et al. 2021), but lower than 0.97% reported by Zhou et al. (Zhou et al. 2020). Additionally, our findings indicated a prevalence lower than 0.97–1.1% reported in the early-onset subgroup of Chinese unselected breast cancer cohorts (Deng et al. 2019; Wu et al. 2020), but higher than the rates of 0.53% in a Japanese study (Momozawa et al. 2018) and 0.68% in a multicenter and international study (Breast Cancer Association Consortium et al. 2022) for the younger subgroup (aged ≤ 50 years).

Approximately 6.4% of the individuals in this study exhibited a positive family history. However, a higher percentage of the enrolled patients had a family history of breast cancer and/or ovarian cancer (8.6% and 13.8%, respectively). This was significantly associated with *PALB2* pathogenic variants in the two aforementioned Chinese studies (Deng et al. 2019; Wu et al. 2020). This may partially explain the lower mutation rates in our early-onset cohort. We observed that patients with a positive family history exhibited a 3-fold higher probability of being the carriers of *PALB2* pathogenic variants than those without a family history (2 vs. 0.69%), although this difference did not reach statistical significance (*p* = 0.178).

In this study, the frequency of *PALB2* pathogenic variants was 1.3% (3/225) among patients aged ≤ 30 years, similar to that of 1%–1.85% in previous studies involving the Chinese (Zhou et al. 2020), Malaysian, and Singaporean populations (Ng et al. 2022). Additionally, we observed an approximately 2-fold occurrence of *PALB2* pathogenic variants in breast cancer patients aged ≤ 30 years compared to those aged 31-40 years (1.3% vs 0.68%), however, the difference was not statistically significant (*p* = 0.398). Zhou JJ et al. reported a significant difference in *PALB2* pathogenic variants between patients aged ≤ 30 years and those > 30 years (*p* = 0.04) in an unselected breast cancer cohort in China (Zhou et al. 2020). The rarity of *PALB2* pathogenic variants may restrict the statistical power of our analysis, and larger scale cohorts of early-onset breast cancer require further assessment to determine whether there is a significant association between age at early onset and *PALB2* pathogenic variants.

*PALB2* pathogenic variants are reported to be significantly associated with TNBC or HR-negative breast cancer in the unselected breast cancer cohorts (Zhou et al. 2020; Breast Cancer Association Consortium et al. 2022). In this study, *PALB2* carriers were more often to be detected in patients with HER2-negative breast cancer than non-carriers (83.3% vs. 70.2%), and HR+/HER2- breast cancer was the predominant molecular subtype among *PALB2* carriers, accounting for 75%, whereas TNBC accounted for only 8.3%. Moreover, patients with HR+/HER2- breast cancer exhibited the highest frequency of *PALB2* pathogenic variants (1.08%) compared to other subtypes, with only 0.41% identified in TNBC. Similar findings were reported in a Chinese hereditary high-risk breast cancer cohort by Ni M et al, with the frequency of *PALB2* pathogenic variants being 1.5% in HR+/HER2- breast cancer and 0.3% in TNBC (Ni et al. 2023). However, the frequency of *PALB2* pathogenic variants in TNBC was 1.9% in the unselected breast cancer cohort (Zhou et al. 2020). This difference in the prevalence of *PALB2* pathogenic variants may be due to the different characteristics of the study populations.

Invasive ductal carcinoma accounted for 75% of all *PALB2* carriers in our study. Besides, two of the 12 (16.7%) *PALB2* carriers were diagnosed with mixed invasive micropapillary carcinoma, an aggressive type of breast cancer with an unfavorable clinical prognosis (Middleton et al. 1999). The mutation frequency of *PALB*2 in patients diagnosed with pure or mixed invasive micropapillary carcinoma was 4.26%, which was approximately 6-fold higher than that in patients diagnosed with invasive ductal carcinoma (0.73%). Given the distinct classification of histological subtypes of breast cancer, only a limited number of studies have documented the risk associated with *PALB2* mutations in invasive micropapillary carcinoma of the breast (Erkko et al. 2007; Breast Cancer Association Consortium et al. 2022). Further independent studies are needed to investigate the association between *PALB2* pathogenic variants and this highly aggressive histological subtype.

The strengths of this study lies in its large sample size of Chinese patients with early-onset breast cancer who were unselected for other predisposing factors, which allows for an objective reflection of the spectrum and clinicopathological characteristics of *PALB2* pathogenic variants in this population. However, this study also has some limitations. First, gene sequencing in this study was conducted using NGS without multiplex ligation-dependent probe amplification (MLPA), which may have led to the omission of some large genomic rearrangements (LGRs). Therefore, the prevalence of *PALB2* variants might be correspondingly underestimated in this study. Some large-scale studies based on European populations reported that pathogenic LGRs account for 2.4-10.3% of all pathogenic *PALB2* variants (Li et al. 2023; Yang et al. 2020) . In contrast, data on Asian populations are limited. A previous Japanese study identified 6 *PALB2* carriers among 568 patients with hereditary breast and ovarian cancer syndrome, and none of whom had LGRs (Kaneyasu et al. 2020). This suggests that the occurrence of LGRs in *PALB2* among Asian breast cancer patients may be very rare. However, considering the limited sample size of this study, further research with larger sample sizes is warranted to validate this observation. In further study, we would included MLPA testing to obtain a more comprehensive understanding of the *PALB2* mutation spectrum in Chinese patients with early-onset breast cancer. Second, the limited number of carriers identified in this study might have diminished the robustness of the statistical analyses, necessitating validation in a larger cohort.

## Conclusion

In summary, the prevalence of germline pathogenic *PALB2* variants was approximately 0.77% in Chinese patients with *BRCA1/2*-negative early-onset breast cancer patients, irrespective of family history. Except for *PALB2* c.1056_1057del (detected twice), all other variants were detected once. Our findings may contribute to the genetic testing strategies for early-onset breast cancer patients in China.

## Data Availability

All data and material relevant to the study are available from the corresponding author on reasonable request.
